# Editorial: Chondrogenic potentials, protocols and mechanisms of mesenchymal progenitor cells

**DOI:** 10.3389/fcell.2023.1289438

**Published:** 2023-09-15

**Authors:** Aaron W. James, Neelima Thottappillil, Bruno Péault, Xinli Zhang

**Affiliations:** ^1^ Department of Pathology, Johns Hopkins University, Baltimore, MD, United States; ^2^ Department of Orthopaedic Surgery, University of California, Los Angeles, Los Angeles, CA, United States; ^3^ School of Dentistry, University of California, Los Angeles, Los Angeles, CA, United States

**Keywords:** mesenchymal stem cells, chondrogenic differentiation, cartilage regeneration, animal models, clinical application

From the pivotal observation of the clonogenic nature of bone-forming cells in bone marrow by Friedenstein (1990); Bianco et al. (2008) to its application in thousands of clinical trials (see ClinicalTrials.gov), the history of mesenchymal stem cell research has remained vivid (Caplan, 1991). Recently, the International Society for Cell & Gene Therapy (ISCT) committee provided a statement to clarify the nomenclature of mesenchymal stem/stromal cells (Viswanathan et al., 2019). Mesenchymal stem/progenitor cells (MSCs) exist in multiple tissues within our body. These cells when dissociated from the native tissue and cultured *in vitro* show the potential to differentiate into multiple mesodermal cell lineages, suggesting applicability to a myriad of tissue regeneration approaches (James et al., 2012). Mesenchymal stem cell-based therapy is used in musculoskeletal repair (James and Péault, 2019; Xu et al., 2021). Even though bone marrow was the first source of MSCs, cells from adipose tissue are presently a major supply of related cells for various applications (Craig et al., 2022). Collectively coined by the term “adipose stromal vascular fraction derived cells”, they constitute stem cells together with endothelial, immune, and other supporting cells (Thottappillil et al., 2023). Many reconstructive and regeneration therapies rely on mesenchymal stem cells, but the exact mechanism underlying the broad term “mesenchymal stem cell-mediated tissue regeneration” is not well understood. The role of MSCs in tissue repair can range from complete restorative of damaged tissue architecture to a growth factor secretor and even to an immune modulator (Scott et al., 2011; Murray and Peault, 2015; Caplan, 2017; Gomez-Salazar et al., 2020). Moreover, mesenchymal progenitors are heterogeneous in their identity and function. One of the common niches for these progenitor cells is the perivascular space of blood vessels, and hence these cells are known as perivascular stem cells or PSCs. PSCs span both pericytes in the microvasculature and adventitial cells around larger vessels (Crisan et al., 2008; Corselli et al., 2013; James and Péault, 2019). Our recent studies have identified a diversity of perivascular progenitors (Ding et al., 2020; Xu et al., 2020; Wang et al., 2020).

Mesenchymal progenitor cells can form lineage-specific cells including chondrocytes (Dominici et al., 2006; Li et al., 2016; Hindle et al., 2017). Although MSCs produce fibrocartilage, not hyaline cartilage, several studies show successful regeneration of articular tissue using MSCs. Our recent reviews compile the role of pericytes in cartilage repair and regeneration (James et al., 2017; Negri et al., 2022). Identification of surface markers helps to predict the chondrogenic differentiation of MSCs and perform their isolation from the heterogenous pool. For instance, receptor tyrosine kinase-like orphan receptor 2 (*ROR2*) was identified as a cell surface marker of MSCs with enhanced capacity for cartilage formation (Dickinson et al., 2017).

The goal of the present Research Topic was to emphasize the chondrogenic potential of mesenchymal progenitor cells in culture, and mechanisms to *in vivo* applications. The Research Topic includes four articles that provide ideas on defining the heterogeneous nature of mesenchymal progenitor cells and efficiently using them for cartilage regeneration.

The original research by Ghabriel et al. aims to identify markers that can be used together with the International Society for Cellular Therapy (ISCT) minimum criteria to define mesenchymal stromal/stem cells. In the present study, the authors carried out a comparative analysis of publicly available single-cell RNA sequencing datasets of MSCs derived from different tissues in both humans and mice. The authors hypothesize that there exists a relationship between protein homeostasis (proteostasis) and mesenchymal stem cell function. Computationally, the study finds six members of proteasomal machinery are promising additional stemness-related markers for accurately validating the identity of MSCs.

Another study by Magallanes et al. focuses on the fate of Gli1+ mesenchymal stem cells post-knee injury in mice. Their results indicate that Gli1+ cells get activated, expand, and differentiate into fibrotic cells in surgically induced knee arthrofibrosis, and significantly contribute to the osteoblast pool during trauma-induced heterotopic ossification. Moreover, the study establishes a novel mouse model for studying post-traumatic knee injury, which can be applied to study other cell populations in the injured joint.

The mini-review by Liu et al. updates the relevance of mesenchymal stem cell therapies to articular cartilage regeneration in large animals. The authors comprehensively present the pros and cons of MSC treatment in large animals ranging from pigs to dogs. They conclude that MSC-based studies for articular cartilage regeneration are to be conducted in either ruminants or pigs, mainly because of anatomical/biomechanical similarity with humans. The mini-review also includes the major tissue engineering strategies for optimized application of MSCs for regenerating cartilage. The authors interestingly point out that there is relevance in the choice of autologous, allogenic, or xenogenic MSCs, which shape the success of cartilage regeneration in large animals.

Another study by Ren et al. directs to the clinical application of adipose stromal vascular fraction (SVF) to treat human knee osteoarthritis. In detail, the article explains the clinical phase I/II trial using MSCs to treat cartilage degeneration in six patients with both knee cartilage defects I-II due to osteoarthritis. Liposuction was performed on the same patient to extract SVF. Postoperatively for 24 weeks, a novel 3D MRI imaging and spatial alignment method was used to evaluate quantitative and qualitative cartilage regeneration in these patients. Conclusively, the authors found that clinical phase I/II application of stromal vascular fraction enhanced cartilage regeneration. The study also provides novel protocols and data for phase-III clinical trials to further test the efficacy of SVF-based cartilage regeneration.

The studies described here contribute to expanding current knowledge on mesenchymal stem cells and cartilage regeneration. Several promising ideas, disease models and protocols were discussed, hoping to improve the health of patients with degenerated cartilage in the future.
